# Comparative Genomics and Metabolomics in the Genus *Nocardia*

**DOI:** 10.1128/mSystems.00125-20

**Published:** 2020-06-02

**Authors:** Daniel Männle, Shaun M. K. McKinnie, Shrikant S. Mantri, Katharina Steinke, Zeyin Lu, Bradley S. Moore, Nadine Ziemert, Leonard Kaysser

**Affiliations:** a Pharmaceutical Biology, Eberhard Karls University Tübingen, Tübingen, Germany; b German Centre for Infection Research (DZIF), Tübingen, Germany; c Interfaculty Institute for Microbiology and Infection Medicine Tübingen (IMIT), Microbiology and Biotechnology, University of Tübingen, Tübingen, Germany; d Department of Chemistry and Biochemistry, University of California, Santa Cruz, California, USA; e Scripps Institution of Oceanography, University of California, San Diego, California, USA; f Skaggs School of Pharmacy and Pharmaceutical Sciences, University of California, San Diego, California, USA; University of California, Berkeley

**Keywords:** *Nocardia*, biosynthetic gene cluster, genome mining, genomics, metabolomics, natural products, siderophores

## Abstract

Using automated genome analysis tools, it is often unclear to what degree genetic variability in homologous biosynthetic pathways relates to structural variation. This hampers strain prioritization and compound identification and can lead to overinterpretation of chemical diversity. Here, we assessed the metabolic potential of *Nocardia*, an underinvestigated actinobacterial genus that is known to comprise opportunistic human pathogens. Our analysis revealed a plethora of putative biosynthetic gene clusters of various classes, including polyketide, nonribosomal peptide, and terpenoid pathways. Furthermore, we used the highly conserved biosynthetic pathway for nocobactin-like siderophores to investigate how gene cluster differences correlate to structural differences in the produced compounds. Sequence similarity networks generated by BiG-SCAPE (Biosynthetic Gene Similarity Clustering and Prospecting Engine) showed the presence of several distinct gene cluster families. Metabolic profiling of selected *Nocardia* strains using liquid chromatography-mass spectrometry (LC-MS) metabolomics data, nuclear magnetic resonance (NMR) spectroscopy, and GNPS (Global Natural Product Social molecular networking) revealed that nocobactin-like biosynthetic gene cluster (BGC) families above a BiG-SCAPE threshold of 70% can be assigned to distinct structural types of nocobactin-like siderophores.

**IMPORTANCE** Our work emphasizes that *Nocardia* represent a prolific source for natural products rivaling better-characterized genera such as *Streptomyces* or *Amycolatopsis*. Furthermore, we showed that large-scale analysis of biosynthetic gene clusters using similarity networks with high stringency allows the distinction and prediction of natural product structural variations. This will facilitate future genomics-driven drug discovery campaigns.

## Abstract (precis)

Our work emphasizes that *Nocardia* represent a prolific source for natural products rivaling better-characterized genera such as *Streptomyces* or *Amycolatopsis*. Furthermore, we showed that large-scale analysis of biosynthetic gene clusters using similarity networks with high stringency allows the distinction and prediction of natural product structural variations. This will facilitate future genomics-driven drug discovery campaigns.

## INTRODUCTION

Bacterial natural products are an important source for small-molecule pharmaceutics. Historically, they have played a major role in what is arguably the greatest success story of modern medicine: the containment of microbial infectious diseases. Nowadays, natural products still represent frequently used lead structures for the development of antibiotic, anticancer, and immunosuppressive agents (1). However, over the last decades, the pharmaceutical industry has shown dwindling interest in classic natural product screening programs. This is primarily due to the increasing rediscovery rate of already known molecules especially in well-investigated bacterial genera such as *Streptomyces*. In recent years, several developments have revived interest in microorganisms as a source of new drug leads. On the one hand, underinvestigated bacterial taxa were shown to comprise prolific producers of novel bioactive chemistry (2). On the other hand, genome sequencing revealed that even in extensively studied bacteria, the molecules isolated so far represent only the tip of the iceberg (3). The biosynthetic capacity of filamentous actinobacteria, such as *Streptomyces*, and myxobacteria, cyanobacteria, and many others, often exceeds 30 or more predicted secondary metabolites per strain. Notably, most of these orphan pathways cannot be associated with known compounds, making them a formidable source for new chemistry and drug leads. However, with more than 400,000 putative gene clusters in the database of the Joint Genome Institute (JGI), the development of bioinformatic tools to assess and prioritize these pathways has become crucial (4). For this purpose, a number of different web-based genome analysis platforms have been established allowing gene-to-molecule predictions of varying accuracy (5). Firm knowledge of the limitations of such approaches is of critical importance to evaluate the novelty of encoded compounds with sufficient confidence.

**FIG 1:**
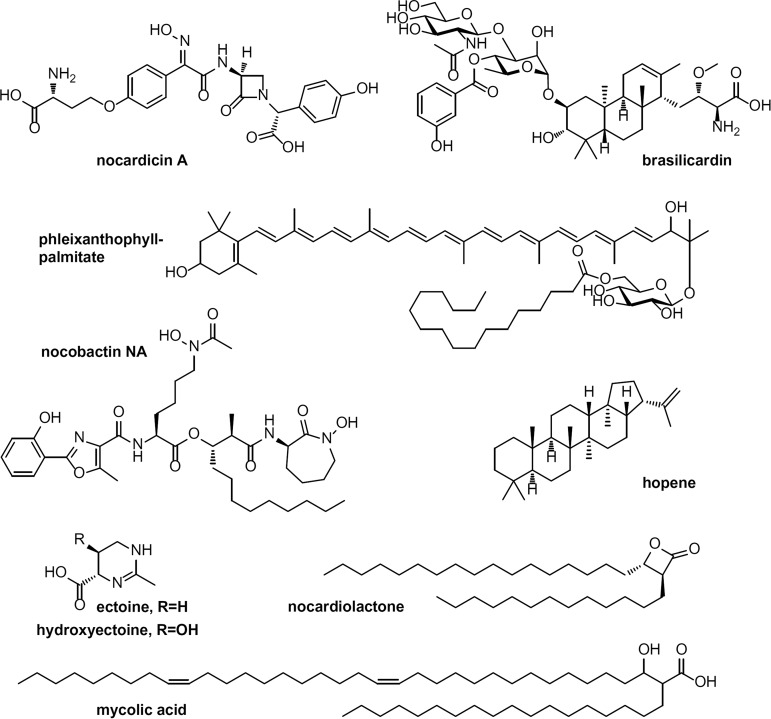
Selected known and predicted compounds from *Nocardia* spp.

Here, we explore the potential of the genus *Nocardia* for the biosynthesis of natural products. Moreover, we use the highly conserved nocobactin pathway to probe thresholds and limits of popular gene cluster analysis tools. *Nocardia* are rod-shaped partially acid-fast actinobacteria of the order *Corynebacteriales*, closely related to *Rhodococcus*, *Corynebacterium,* and *Mycobacterium*. They are able to form branching, filamentous mycelia that can fragment into bacteroid nonmotile elements. *Nocardia* are ubiquitous saprophytes often found in organic-rich soil, but they are most prominently known as opportunistic pathogens. Nocardiosis is an infectious disease affecting the immunocompromised with a high mortality rate, but it has also been reported for immunocompetent patients (6). Commonly, *Nocardia* cause localized cutaneous or pulmonary infections depending on the nature of exposure to the pathogen. From here, the bacteria may disseminate, leading to systemic forms of nocardiosis and severe brain infections. The pathogenicity of *Nocardia* has been extensively studied by Beaman and others (7). Still, major aspects are poorly understood, including the role of virulence factors in strain-specific development and characteristics of the disease. Over the last decades, *Nocardia* have also served as the occasional source for natural products (Fig. 1) (8–11). However, in comparison to other genera of the phylum, the chemistry of *Nocardia* is vastly underexplored. Notably, the preliminary analysis of the first seven available *Nocardia* genomes by Komaki et al. in 2014 showed a diverse repertoire of nonribosomal peptide synthetase (NRPS) and type I polyketide synthase (PKS) pathways (12). The authors found that the content of respective gene clusters varies considerably between different clinically relevant *Nocardia* pathogens, ranging from 12 such pathways in Nocardia farcinica IFM 10152 to 30 in some investigated Nocardia brasiliensis strains (IFM 10847 and HUJEG-1). NRPS and type I PKS are multimodular biosynthetic machineries in which every module is responsible for the incorporation of a building block by assembly line logic. They are important driving forces for the generation of chemical diversity and structural complexity in bacterial natural products. Other relevant classes of secondary metabolites include type II and type III polyketides, terpenoids, ribosomally synthesized and posttranslationally modified peptides (RiPPs), alkaloids, and nucleosides. In the current report, we present a comprehensive analysis of the biosynthetic capacity of the genus *Nocardia* based on 103 published genomes. To this end, we used an integrated workflow that combines the most popular gene cluster analysis tool antiSMASH and the recently developed BiG-SCAPE algorithm to generate sequence similarity networks (13, 14). With this setup, we observed tremendous potential for the production of bioactive small molecules rivaling *Streptomyces* in number and diversity. Several biosynthetic pathways were found to be conserved throughout the genus including a gene cluster for the formation of nocobactin-type siderophores. Using this pathway as a proxy, we probed different stringencies to construct the sequence similarity networks and correlated the generated gene cluster families (GCFs) with a mass spectrometry (MS) metabolic network. Strikingly, by applying a BiG-SCAPE threshold of 70%, we were able to produce GCFs that could be assigned to the synthesis of structurally distinct groups of nocobactin derivatives.

## RESULTS AND DISCUSSION

### Phylogeny and biosynthetic potential of the genus *Nocardia*.

**FIG 2:**
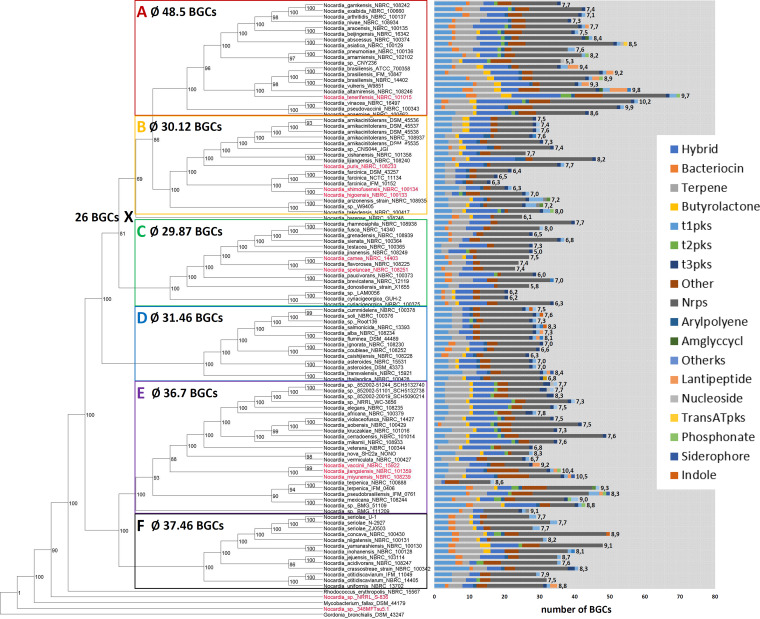
*Nocardia* phylogeny and BGC distribution. Maximum likelihood tree of 103 members of the genus *Nocardia* based on 63 concatenated housekeeping genes identified with autoMLST (see Fig. S2 and Table S3 at https://doi.org/10.5281/zenodo.3784407). Ultrafast bootstrap values were calculated using 1,000 replicates. Numbers next to the bars (predicted BGCs by antiSMASH) represent genome sizes (in megabase pairs). Average numbers of BGCs per clade are shown. X labels a distinct branch formed by a single *Nocardia* strain, Nocardia harenae NBRC 108248.

A search in public databases such as NCBI and JGI found 103 nonredundant genome sequences from organisms named *Nocardia*, as of 2017. The genomes had an average of 147 contigs (1 to 615) with an estimated completeness of at least 98.24%, as analyzed by checkM (see Table S20 at https://doi.org/10.5281/zenodo.3784407). In order to obtain an overview about the diversity of the genus and evolutionary relationships within the genus, we built phylogenetic trees using the web tool autoMLST (15). To this end, 63 conserved housekeeping genes with neutral *dN*/*dS* (ratio of nonsynonymous to synonymous evolutionary changes) values were identified by autoMLST and used for the concatenated alignment as a basis for tree building. The resulting tree (Fig. 2) shows six major clades (A to F) within the genus and one distinct branch (X) formed by the single strain Nocardia harenae NBRC 108248. Interestingly, two strains formed a clade outside the outgroups, indicating that they do not belong to the genus *Nocardia*. MASH analysis (16) to estimate the average nucleotide identity (ANI) showed that both strains, named *Nocardia* sp. strain NRRL S-836 and *Nocardia* sp. strain 348MFTsu5.1, are more similar to Lentzea guizhouensis and Williamsia muralis NBRC 105860 with an ANI of 91.12% and 78.91%, respectively, thus providing further evidence that these strains are incorrectly assigned and do not belong to the genus *Nocardia* (17). Furthermore, these results were supported by checkM analysis, as both strains fall into different lineage markers. We therefore did not include these two strains in further analysis. From the 101 remaining analyzed *Nocardia* strains, 50 are clinical isolates, seven have been isolated as animal and seven as plant pathogens, and 31 are derived from environmental sources. *Nocardia* of all origins are evenly spread across the six phylogenetic clades, suggesting that pathogenicity of *Nocardia* strains is not reflected by evolutionary relationship (see Fig. S30 at https://doi.org/10.5281/zenodo.3784407). To assess the biosynthetic potential of the strains, we performed computational genome mining analysis using the program antiSMASH (13). All analyzed genomes contain a variety of biosynthetic gene clusters (BGCs) belonging to the major biosynthetic classes (Fig. 2). On average, a *Nocardia* genome features 36 BGCs and a predicted overall chemical/metabolic diversity of 35% NRPs, 18% PKs, 13% terpenoids, 12% hybrids, 2.2% bacteriocins, 2.2% arylpolyenes, 2% butyrolactones, 1.1% RiPPs, and 14.5% others. Gratifyingly, the number of identified pathways did not correlate with the number of contigs per genome (*R*^2^ = 0.28; see Fig. S32 at https://doi.org/10.5281/zenodo.3784407). Although this warrants a certain degree of robustness to our data, actual BGC numbers might vary slightly, as antiSMASH results were not reinspected for broken or merged clusters. Interestingly, we could observe a correlation between the phylogenetic clade and BGC abundance. Clades A, E, and F contain on average more BGCs than clades B and C. However, even in strains with relatively low BGC numbers, at least 16 BGCs were detected. Similar to their sister genus *Rhodococcus*, the most prevalent BGCs in *Nocardia* are NRPSs (18). A positive correlation was found between the number of biosynthetic gene clusters in each strain predicted by antiSMASH and the respective genome size (see Fig. S1 at https://doi.org/10.5281/zenodo.3784407). Overall, the established biosynthetic capabilities of *Nocardia* strains are comparable to those of *Streptomyces* and *Amycolatopsis*, based on recently reported diversity plots (18–20).

### Genomic features of clinically relevant *Nocardia* strains.

*Mycobacterium* and *Rhodococcus* pathogenic strains generally have smaller genomes as well as fewer and less diverse BGCs than the rest of the respective genera (21). The opportunistic nature of nocardiosis, the high diversity of causative species, and the insufficient knowledge on *Nocardia* virulence factors makes the distinction of pathogenic and nonpathogenic strains difficult. Accordingly, we were not able to observe a correlation between the genome size or BGC content and the different sources of isolation (see Fig. S30 at https://doi.org/10.5281/zenodo.3784407). It is fair to assume that in principle, many *Nocardia* strains can act as facultative pathogens. Nevertheless, some *Nocardia* species are reported more often from nocardiosis patients than others, i.e., N. asteroides, N. cyriacigeorgica, N. brasiliensis, N. abscessus, N. farcinica, N. nova, and N. otitidiscaviarum, further referred to as group 1 pathogens (6, 22–24). Indeed, the genomes of these pathogens are on average slightly smaller than the rest of the genus (7.4 Mbp versus 7.8 Mbp) and contain fewer BGCs (32.5 versus 36.5). A remarkable exception are the different strains of N. brasiliensis with genomes of 9.4 (ATCC 700358), 9.2 (IFM 10847), and 8.9 (NBRC 101014) Mbp in size and an outstanding chemical potential with 49, 56, and 49 predicted BGCs, respectively. It is notable that *N. farcinica* IFM 10152, a clinically prevalent group 1 pathogen, with 6.3 Mbp contains the smallest genome and the lowest number of predicted BGCs (16 BGCs) of all *Nocardia*. Intriguingly, a majority of these clusters are conserved in other *Nocardia* spp., sometimes throughout the genus (see Table S22 at https://doi.org/10.5281/zenodo.3784407). It could be speculated that the BGCs found in *N. farcinica* represent some version of a minimal secondary metabolome required to support both a lifestyle as a soil-dwelling saprophyte and as a successful opportunistic pathogen. A preliminary survey of putative *Nocardia* virulence factors such as catalases and superoxide dismutases showed no significant trend regarding number and identity of respective genes in group 1 strains versus the rest of the genus (see Table S21 at https://doi.org/10.5281/zenodo.3784407). This could suggest that strain-specific pathogenicity is mainly driven by other characteristics.

### Metabolic diversity and gene cluster families in *Nocardia*.

**FIG 3:**
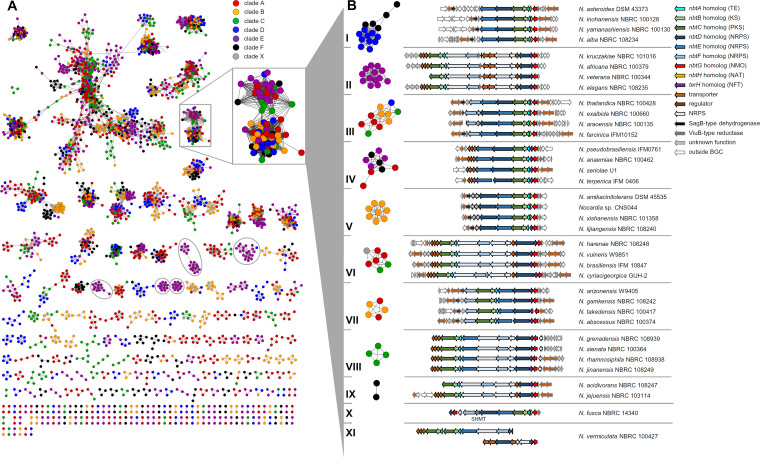
BiG-SCAPE sequence similarity network (SSN) and nocobactin-like subfamilies visualized in Cytoscape. (A) SSN (*c* = 0.5) of *Nocardia* spp. Each node represents one BGC identified by antiSMASH 4.0. Colors represent phylogenetic clades A to F and X. Clade-specific GCFs are highlighted. (B) SSN (*c* = 0.7) of nocobactin-like BGCs. Representative pathways of different subfamilies (I to IX). Two singletons (X and XI) are shown. Abbreviations: TE, thioesterase; KS, ketosynthase; PKS, polyketide synthase; NRPS, nonribosomal peptide synthetase; NMO, *N*-monooxygenase; NAT, *N*-acetyltransferase; NFT, *N*-formyltransferase; SHMT, serine hydroxymethyltransferase.

**FIG 4:**
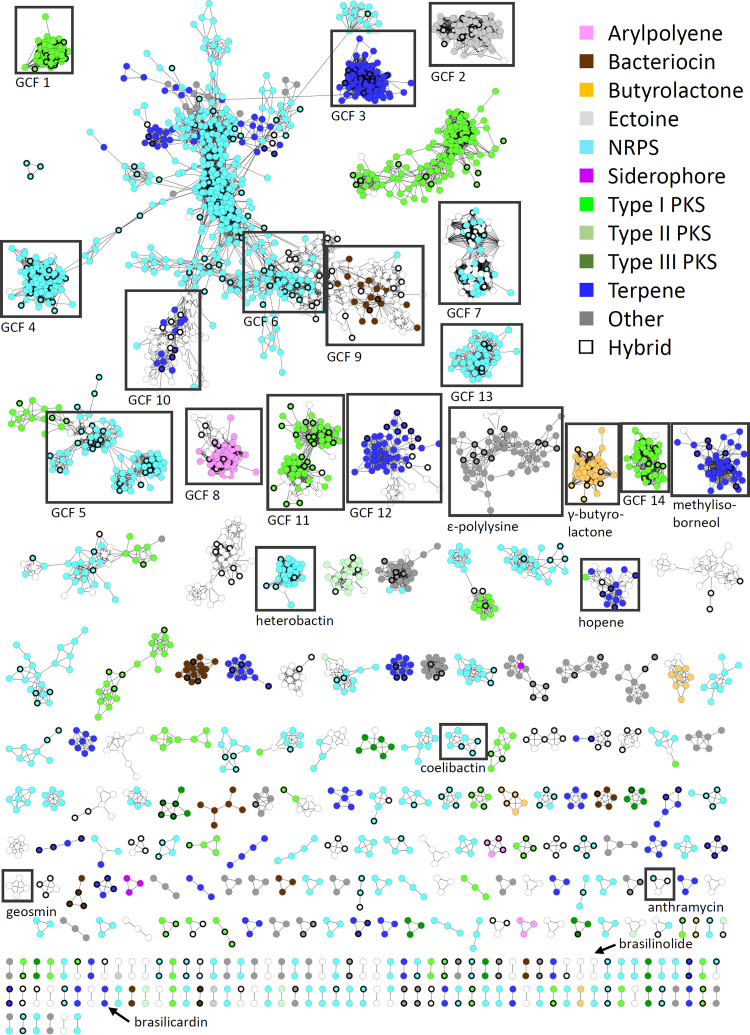
BiG-SCAPE cluster type sequence similarity network of all BGCs from *Nocardia* strains found with antiSMASH 4.0. Colors show cluster types from *Nocardia* BGCs. Colorless nodes are hybrid BGCs found in *Nocardia*. Bold node borders refer to group 1 pathogens.

To assess the diversity of BGCs in the genus in more detail, we performed a network analysis using the BiG-SCAPE algorithm. BiG-SCAPE employs Pfam composition similarity to calculate BGC distances that takes a weighted combination of the Jaccard index, adjacency index, and domain sequence similarity score into account (14, 25). The generated sequence similarity network with a default similarity score cutoff of *c* = 0.5 (equals threshold of 50%) consisted of 2,836 BGCs as unique nodes and 22,455 edges (Fig. 3 and 4). Altogether, 258 independent gene cluster families (GCFs) were formed with two or more nodes and 750 singletons. Notably, several large superclusters are visible, which are composed of subclusters with apparently different biosynthetic features (Fig. 4). These superclusters could not be satisfyingly resolved using higher similarity cutoffs or different alignment modes. Such problems have been reported from other comparative genome analyses and attributed to fragmented genome assemblies and deficient separation of individual BGCs by antiSMASH. (18). Indeed, manual inspection of the *Nocardia* BGC similarity network revealed a number of distinct subclusters we considered additional GCFs (Fig. 4). Next, we wanted to assess whether the clinically most relevant *Nocardia* species exhibit a characteristic secondary metabolism and conducted a number of statistical analyses. Boxplots along with *t* test indicate that the BGC class frequency distribution between group 1 and other *Nocardia* genomes is insignificant (see Fig. S33 at https://doi.org/10.5281/zenodo.3784407). Moreover, principal-component analysis (PCA) plots show that the GCF presence/absence information is insufficient for clustering the *Nocardia* genomes into separate groups based on clinical annotations (group 1; see Fig. S34 at https://doi.org/10.5281/zenodo.3784407). Overall, it appears that none of the BGCs are significantly more abundant or even specific to prevalent *Nocardia* pathogens in comparison to the rest of the genus. However, correlation can be observed between phylogenetic clades and BGC distribution within the sequence similarity network (Fig. 3). In particular, strains from clade E feature a number of clusters that are not found in bacteria from other clades, including pathways for terpenoid, NRPS, and PKS biosynthesis. For example, a gene cluster with high similarity to hopene biosynthesis is found in all *Nocardia* of clade E and otherwise only in Nocardia transvalensis NBRC 15921. Hopanoids (Fig. 1) are pentacyclic triterpenoid components of membranes from diverse prokaryotes (26). They have stabilizing functions similar to sterols in eukaryotes and might be of use to members of clade E in specific environments. In contrast, BGCs of *Nocardia* spp. attributed to clade F do not form a single characteristic GCF with representatives from more than seven strains.

A number of GCFs comprise clusters, which are highly abundant in the investigated *Nocardia* genomes (Fig. 4). This includes several GCFs that represent pathways with mono-modular type I PKS-like ketosynthases, i.e., GCF 1, 11, and 14, potentially involved in the modification of fatty acid precursors. For example, GCF 1 represents a highly conserved cluster found throughout the genus, which likely directs the synthesis of mycolic acids. Mycolic acids (Fig. 1) are characteristic and essential cell wall components of mycobacteria and related genera, e.g., *Corynebacterium*, *Rhodococcus*, *Gordonia,* and *Nocardia* (27). Depending on the producing organism, these molecules differ in chain length and degree of unsaturation. *Nocardia* mycolic acids usually consist of a C_32-40_ β-hydroxy chain and a C_12-18_ α-alkyl side chain. A typical cluster of GCF 1 encodes a homolog of Pks13, the essential condensing enzyme to form mycolic acid from two fatty acyl coenzyme A (acyl-CoA) precursors (28). It contains additional genes that are assigned to the biosynthesis and transfer of arabinose and galactose. Together, these enzymes presumably participate in the assembly of the *Nocardia* mycolyl-arabinogalactan-peptidoglycan complex. Nocardiolactone (Fig. 1) is a fatty acid derivative with similarity to lipstatin that has been isolated from different *Nocardia* strains (29). Recently, Robinson and coworkers reported on the identification of the nocardiolactone gene cluster featuring a β-lactone synthetase (30). β-Lactone-containing natural products are potent inhibitors of diverse hydrolytic enzymes, and their biosynthesis has drawn a lot of attention (31–33). The lower subcluster of GCF 11 contains 36 homologous nocardiolactone BGCs of which six have been initially assigned by antiSMASH as putative lipstatin-type pathways (Fig. 4).

The BGCs that form GCF 2 are found in all *Nocardia* genomes and encode enzymes for the synthesis of ectoine (Fig. 1), a compatible solute which stabilizes biopolymers (DNA, proteins, etc.) under extreme conditions (34). The capability to produce ectoine or 5-hydroxyectoine is not uncommon to actinobacterial genera such as *Streptomyces* and *Mycobacterium* (35, 36). The strict conservation of this pathway in *Nocardia* seems to reflect a general requirement of all members of the genus to be able to function under osmotic stress. It was recently shown that the ectoine BGC is similarly conserved in the sister genus *Rhodococcus* (18).

*Nocardia* are known to produce colorful pigments ranging from yellow to red, but a systematic analysis of the carotenoid content in this genus has not been conducted (7). GCF 3 summarizes pathways that are likely responsible for the formation of a carotenoid of unknown structure. The BGC is highly conserved in all *Nocardia* genomes. It encodes polyprenyl synthase (CrtE), phytoene synthase (CrtB), and phytoene desaturase (CrtI) homologs for the production of lycopene. An obvious candidate for a cyclase is not found in the cluster. However, a putative lycopene cyclase present in all *Nocardia* species is the main feature of the conserved terpene GCF 10. Together, both BGCs could direct the concerted biosynthesis of mono- or bicyclic carotenes. Further modifications may involve the attachment of a glycosyl group similar to sioxanthin (37). Such monocyclic carotenoid glycosides and their esters (Fig. 1) have been isolated from Nocardia kirovani and different *Rhodococcus* species (38, 39). Arylpolyenes are another common class of pigments and generated by an unusual type II PKS system. These molecules are mostly known from Gram-negative gammaproteobacteria and bacteroides (25). A pathway with signature motifs for the synthesis of aryl polyenes is widespread in *Nocardia* (82 of 101 genomes) and clustered in GCF 8.

The gene clusters organized in GCF 4 are also found in all *Nocardia* species. Here, a single NRPS with four modules is colocated with a putative *N*-acyltransferase and two transporters. The produced peptide likely consists of an N-terminal serine and three additional amino acids. The three residues cannot be predicted with certainty from the respective incorporating A domains but presumably feature aliphatic or aromatic side chains (see Table S4 at https://doi.org/10.5281/zenodo.3784407). Two other conserved NRPS pathways could be identified. One of them (GCF 5) consists of a trimodular NRPS gene with epimerization (E) domains encoded in the first module and in the terminal module. A peptide sequence could not be predicted on the basis of the A domains. This BGC is present in 75 out of 101 *Nocardia* genomes. Similarly conserved is a large NRPS assembly line of different size represented by GCF 6. The enzyme complex may contain 7 to 13 modules, but the gene cluster environment is invariant and always encodes a putative nitropropane dioxygenase, a metallopeptidase, an esterase, an epimerase, and an enoyl-CoA dehydratase.

Linocin M18 has been described as a secreted bacteriolysin (formerly type III bacteriocin) from Brevibacterium linens M18 (40). Similar proteins were found in various other bacteria, including Mycobacterium tuberculosis and Burkholderia cepacia complex. They could be associated with pathogen-host cell attachment and immunogenicity (41, 42). Moreover, linocins have been shown to form multimeric microcompartments (43). A respective orthologous gene is encoded in all *Nocardia* genomes and summarized in GCF 9.

### The gene cluster family of nocobactins.

**FIG 5:**
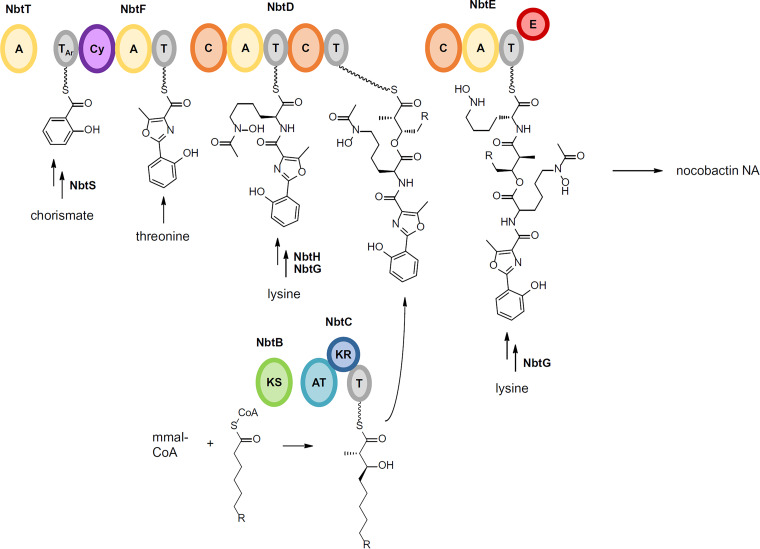
Model for nocobactin NA biosynthesis. NRPS/PKS domains: adenylation (A), thiolation (T), heterocyclization (Cy), condensation (C), epimerization (E), ketosynthase (KS), acyltransferase (AT), ketoreductase (KR), aryl carrier protein (T_Ar_), methylmalonyl CoA (mm-CoA).

Another highly abundant BGC family (GCF 7) is found in 92 of 101 *Nocardia* genomes and encodes a conserved hybrid NRPS/PKS pathway annotated by antiSMASH as putative nocobactin NA BGC (>50% homology; Fig. 3A). Nocobactin NA (Fig. 1) was first reported in 1974 as a UV-active siderophore from cultures of N. asteroides by Ratledge and Snow (44). Nocobactins were subsequently found in other *Nocardia* spp., but not in *Rhodococcus* (45). A number of derivatives have been isolated since, including the amamistatins, nocardimicins, brasilibactin, formobactin, and terpenibactins (46–50), which all share a hydroxyphenyloxazoline/-oxazole moiety, a *N*-acetylated or *N*-formylated *N*-hydroxylysine that is linked via an ester moiety to a long-chain α-methyl, β-hydroxy fatty acid, and a C-terminal *N*-hydroxy α-amino ε-caprolactam ring. The biosynthetic pathway for nocobactin NA reported from *N. farcinica* IFM 10152 involves a nonlinear assembly line consisting of three NRPS modules and one PKS module (Fig. 5) (51). NbtF (T-cyclization domain [Cy]-A-T) is proposed to catalyze the condensation and heterocyclization of salicylic acid and threonine with the help of the stand-alone A-domain NbtT. NbtD (C-A-T-C-T) activates and adds lysine, or a derivative, to the growing chain. NbtH and NbtG, either before or after incorporation of the lysine moiety, likely direct *N*-acylation and *N*-hydroxylation, respectively. NbtB (KS) and NbtC (AT-ketoreductase [KR] domain-T) are proposed to be responsible for the activation of a fatty acid which is extended by Claisen-type condensation with methylmalonyl-CoA and subsequently reduced at the β-position. The incorporation of the fatty acyl moiety by ester linkage is probably mediated by NbtD before NbtE (C-A-T-E) attaches another lysine. Epimerization of this amino acid to the d isomer is followed by *N-*hydroxylation and formation of the ε-caprolactam. Notably, the nocobactin pathway is split in two gene clusters, which are located 180 kb apart on the *N. farcinica* IFM 10152 chromosome. Cluster I contains the majority of the biosynthetic genes, whereas cluster II codes for NbtT and a putative salicylate synthase, NbtS. This splitting is reflected in the BiG-SCAPE similarity network by GCF 13 that is annotated by antiSMASH as mycobactin-like with 30% similarity. In fact, this GCF contains a small pathway of three conserved genes with high homology to *nbtT*, *nbtS,* and *nbtF*. It appears that nocobactin cluster II (which is not deposited in the MIBiG database) is recognized bioinformatically only if the NRPS module NbtF is encoded. Indeed, all strains containing such a cluster II variant lack an *nbtF* homolog in their respective nocobactin cluster I. This pathway organization is shared by almost 45% of *Nocardia* spp. The widespread occurrence of the nocobactin NA-like pathway in *Nocardia* genomes indicates a constitutive role for this siderophore in the biology of the genus. Structurally and biosynthetically, the nocobactins are closely related to the mycobactins which have similar prominence in mycobacteria (52). Both compound classes differ in the chain length of their acyl substituents. Mycobactins feature a long-chain fatty acid attached to the *N*-hydroxylysine moiety instead of the formyl/acetyl group in nocobactin-like molecules and a central 3-hydroxybutyrate group. They are the quintessential siderophores for the genus *Mycobacterium* and are produced by all species except M. paratuberculosis. These molecules exist typically as membrane-bound iron scavengers but can also be produced in a more polar form as extracellular carboxymycobactins (53). Mycobactins of both variants are important for the lifestyle of pathogenic mycobacteria and have been shown to play a fundamental role in the survival and growth of M. tuberculosis in human macrophages (54, 55). It is tempting to speculate that nocobactins may have a similar function as virulence factors in the progression of nocardiosis. The first evidence for this theory was obtained by Hoshino et al. studying Δ*nbtE* deficient mutants in infection assays with the macrophage-like cell line J774A.1 (51).

To assess the metabolic diversity of nocobactins in the genus *Nocardia*, we analyzed the composition of the BGC family generated by BiG-SCAPE in more detail. Notably, the nocobactin GCF obtained with a threshold of 50% shows two prominent subclusters (Fig. 3A). Both subclusters are characterized on a genetic level by the exclusive presence of a gene for an *N*-acetyltransferase (NbtH homolog) or an *N*-formyltransferase. This finding suggests that the subdivision of the nocobactin GCF reflects the production of structurally different derivatives with either an acetyl or formyl group attached to the *N*-hydroxylysine residue. From the reported nocobactin-like siderophores, nocobactin NA and nocardimicins A to F feature an acetyl moiety, whereas nocardimicins G to I, formobactin, brasilibactin, amamistatins, and terpenibactins feature a formyl moiety. A candidate enzyme for this reaction, TerH, has been identified in the terpenibactin pathway (50). Further refinement of the BiG-SCAPE similarity network with a threshold of 70% resulted in the segmentation of the nocobactin cluster I GCF to nine independent families of more than two BGCs (further referred to as nocobactin-like BGC subfamilies) and nine singletons (Fig. 3B). Interestingly, the composition of these new subfamilies correlates to some degree with the phylogenetic clades observed by autoMLST analysis. Subfamilies II, V, VIII, and IX consist solely of pathways from clades E, B, C, and F, respectively. Subfamilies I and VII are dominated by clades D and F and clades A and B, respectively. Again, the absence or presence of NbtH/TerH homologs is a major discriminating factor that determines these subfamilies. Subfamilies I and III carry a gene that encodes an NbtH, and subfamilies II, IV, VI, and VII to IX carry a gene that encodes a TerH homolog. Only subfamily V comprises pathways that contain either an *nbtH* or *terH* gene. Additionally, splitting of subfamilies is based on the clustering of the *nbtF* homolog. Subfamilies II, III, VI, VII, VIII, and IX exhibit a putative *nbtF* gene in cluster I, whereas in subfamilies I, IV, and V, *nbtF* is colocated with cluster II. Further characteristic family features are additional and/or alternative NRPS modules in subfamilies II, VI, VIII, and IX. Here, the nocobactin-like pathways have a gene that encodes a discrete C-T didomain protein, probably a functional substitute to the missing terminal C-T domains of the NbtD homolog, and another NRPS enzyme with a C-A-T-E-C domain structure. The specificity of the corresponding A domain could not be predicted bioinformatically. It is worth mentioning that the previously reported nocobactin-type molecules exhibit diverse stereochemical configurations. Nocobactin NA features a central acylated *N*-hydroxy l-lysine and a terminal *N*-hydroxy d-lysine 1,6-lactam. In contrast, amamistatin A incorporates d- and l-lysine and brasilibactin A incorporates d- and d-lysine derivatives at these positions, respectively (51, 56, 57). Possibly, the production of different stereoisomers might rely on the presence of additional E-domain-containing NRPS enzymes as found in subfamilies II, VI, VIII, and IX. Moreover, in subfamilies VI and VIII, the PKS enzymes with NbtC homology are extended by a *C*-methyltransferase domain to an AT-KR-T-cMT module with predicted AT domain specificity to methylmalonyl-CoA (mmal-CoA). Notably, formobactin A and the amamistatins contain a rare α-dimethylated fatty acid which could derive from such a biosynthetic setup. It is also worth mentioning that the colocalization of a gene for a putative SagB-type dehydrogenase is characteristic for the clusters subsumed in subfamilies I, III, V, and VII. SagB is involved in the biosynthesis of streptolysin S toxin by catalyzing the oxidation of oxazoline and thiazoline heterocycles to form oxazole and thiazole ring structures (58). Interestingly, both heterocyclic variants exist in nocobactin-type siderophores, with oxazole moieties in nocobactin NA, formobactin, the amamistatins and nocardimicins A to F, and oxazolines in brasilibactin A, nocardimicins G to I, and the terpenibactins. The established subfamilies can also be distinguished based on the association of the pathways with transporter genes. In subfamilies II, IV, VI, VIII, and IX, such functionalities are encoded in the same operon as the biosynthetic genes. In subfamilies I, III, V, and VII, transporters are encoded immediately adjacent to the clusters. Nine gene clusters were not clustered with the other subfamilies. One of these pathways (X), found in Nocardia fusca NBRC 14340, contains homologs to all genes of the nocobactin NA gene cluster. Additionally, it features a second copy of *nbtF*, a *terH* homolog, a gene for a discrete A domain and a putative serine hydroxymethyltransferase gene. Another singleton cluster (XI) is encoded in Nocardia vermiculata NBRC 100427 exhibiting two copies of an *nbtABC*-like operon but lacking *nbtD*, *nbtG,* and *nbtH*/*terH*-like genes. The missing homologs are located in an independent cluster together with genes for a second NRPS C-A-T-E-C module and several transporters.

### Nocobactin-like molecules produced by different *Nocardia* strains.

**FIG 6:**
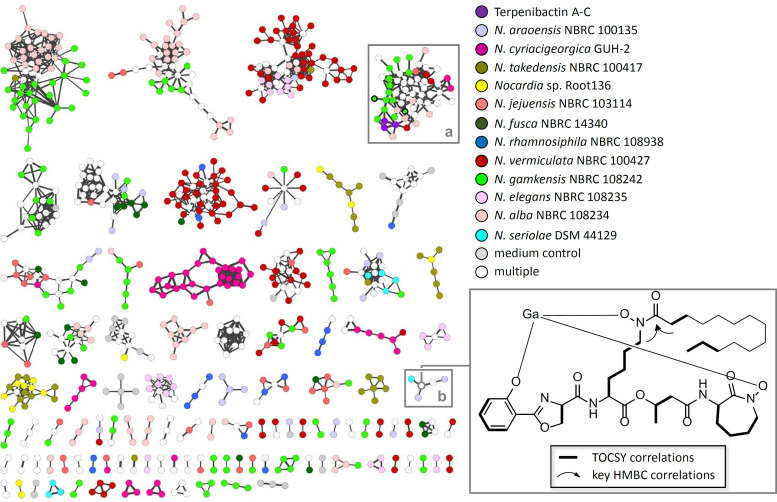
Molecular MS/MS network of culture extracts from selected *Nocardia* strains. The color of the node indicates the source of the ions. Bold node borders indicate putative carboxynocobactins. Molecular families containing putative siderophores a (nocobactin-type) and b (mycobactin-type) are highlighted by gray framed boxes. The structure of the identified mycobactin-type compound from *N. araoensis* NBRC 100135 with an exact mass of 773.4569 Da and *m/z* 840.3669 [M-2H+Ga]^+^ is shown in complex with gallium.

**FIG 7:**
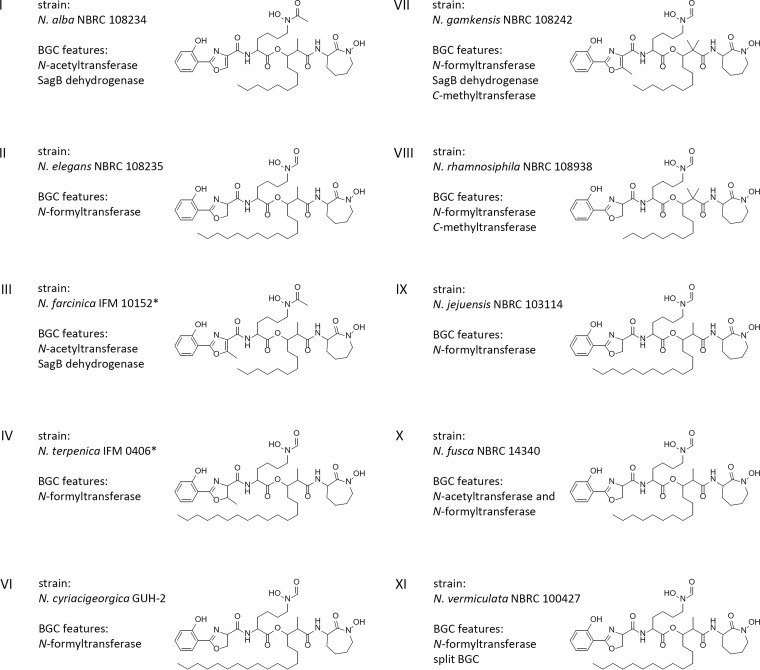
Production of nocobactin-type siderophores by selected *Nocardia* strains from subfamilies I to XI. Representative compounds are depicted. Structures shown in I, II, and VI to XI were predicted from HRMS and MS/MS experiments. An asterisk indicates that the structures shown in III and IV have been adopted from literature (50, 51).

Clearly, the different families obtained from the BiG-SCAPE similarity network are composed of pathways with characteristic biosynthetic functionalities. We next wanted to inquire how these distinct cluster organizations correspond to structural diversity in the produced nocobactin-type siderophores. Therefore, we examined the intracellular and membrane-associated culture fractions from a selection of *Nocardia* strains representative of the identified nocobactin-like BGC subfamilies (see Table S19 at https://doi.org/10.5281/zenodo.3784407). The culture extracts were subjected to positive ion mode liquid chromatography-mass spectrometry (LC-MS) analysis. To stabilize molecules with metal-chelating properties and facilitate their identification, we supplemented the samples with Ga_2_(SO_4_)_3_ under reducing conditions. This treatment resulted in the almost complete equipment of metal-free siderophores with Ga(III) and largely replaced Fe(III) in already loaded compounds. The generated gallium complexes could be detected by mass spectrometry via their distinct isotopic pattern. The tandem mass spectrometry (MS/MS) spectra obtained from data-dependent acquisition experiments were used to construct a molecular network with the help of the GNPS (Global Natural Product Social Molecular Networking) platform (59). For an internal reference, we included pure samples of the recently discovered terpenibactins in our analysis (see Table S5 at https://doi.org/10.5281/zenodo.3784407) (50). The resulting network of ionizable metabolites from 12 *Nocardia* strains together with the terpenibactins represented 3,567 nodes and 4,177 edges (Fig. 6). At a cosine value of 0.6, it forms 108 clusters of two or more nodes and shows 2,470 singletons. The observed clusters likely represent molecular families of compounds with similar chemical structures. The terpenibactins clustered with one molecular family containing 21 putative metabolites of seven analyzed *Nocardia* strains. A closer inspection showed that this association is based on the consistent fragmentation of the terpenibactins along the ester linkage between the modified lysine residue and the β-hydroxy fatty acid moiety. This produces characteristic fragment ions of *m/z* 460.0621 (major), *m/z* 414.0552 (major), and *m/z* 442.0477 (minor) for terpenibactin A (see Fig. S4 at https://doi.org/10.5281/zenodo.3784407). A similar pattern can be observed for terpenibactin B and terpenibactin C with deviating fragment ions containing the variable fatty acyl moiety (see Fig. S3 at https://doi.org/10.5281/zenodo.3784407). Additional prominent fragments of *m/z* 145.1 and *m/z* 171.08 are found in most MS/MS spectra and likely represent the caprolactam moiety. Using these results as fingerprints, we interpreted the other spectra clustered with the terpenibactins. In consideration of the biosynthetic information deduced from the gene clusters and the already known chemical diversity, we postulated possible structures for each of the produced nocobactin derivatives (Fig. 7; see Fig. S5 to S21 and Tables S6 to S15 at https://doi.org/10.5281/zenodo.3784407). The first thing to note is that representatives of all investigated subfamilies seem to synthesize molecules with only minor structural differences to nocobactin NA. The predicted compounds all comprise a phenoxazoline/-oxazole, an acylated *N*-hydroxylysine, a hydroxy fatty acid, and a caprolactam moiety. The integrity of this core structure was independent of the composition of the respective BGCs, e.g., the presence of additional NRPS genes (subfamilies II, VI, VIII, and IX), duplication of the PKS genes (subfamily XI) or absence of the *nbtF* homolog in cluster I (subfamilies I, IV, V, and X). However, the MS/MS characteristics of the different identified nocobactin-type molecules are in accordance with the presence of *nbtH* (*N*-acetyltransferase) or *terH* (*N*-formyltransferase) homologs and PKS *C*-methyltransferase domains in their respective gene clusters. This supports our bioinformatic predictions that generally pathways from subfamilies I, III, and V produce acetylated nocobactin derivatives and pathways from subfamilies II, IV, and VI to IX produce formylated nocobactin derivatives. Strikingly, colocation of a *sagB*-like dehydrogenase gene with the nocobactin biosynthetic pathway in subfamilies I, III, and VII led to the almost exclusive production of molecules with a predicted phenoxazole moiety. The absence of such a gene in subfamilies II, IV, VI, and VIII to XI, on the other hand, correlated with the accumulation of phenoxazoline-containing compounds. This strongly indicates that the SagB-type dehydrogenase is involved in the oxidation of the oxazoline ring in the biosynthesis of this class of siderophores. Our genomics-to-metabolomics analysis of Nocardia alba NBRC 108234 (subfamily I) suggests that this strain synthesizes nocobactin-type siderophores with a nonmethylated oxazole moiety and a *N*-acetyl group (Fig. 7). This closely resembles the structures of the already known nocardimicins A to F. Indeed, exact mass and MS/MS spectra of ions with *m/z* 796.3043 [M-2H+Ga]^+^, *m/z* 824.3356 [M-2H+Ga]^+^, and *m/z* 852.3669 [M-2H+Ga]^+^ perfectly match those of nocardimicins A, B, and D, respectively (see Fig. S18 to S20 at https://doi.org/10.5281/zenodo.3784407). In addition, we would postulate that Nocardia elegans NBRC 108235 (subfamily II) and Nocardia jejuensis NBRC 103114 (subfamily IX) produce *N*-formylated nonmethylated oxazoline-containing molecules with MS characteristics identical to those of nocardimicin G and nocardimicin H (see Fig. S15 and S16 at https://doi.org/10.5281/zenodo.3784407). Similar molecules are produced by *N. cyriacigeorgica* GUH-2, the representative of subfamily VI. This subfamily also comprises the three BGCs found in N. brasiliensis strains ATCC 700358, IFM 10847, and NBRC 14402, respectively. It is noteworthy that a nocardimicin analog, brasilibactin A, has previously been isolated from this species (48). Closer inspection of the N. cyriacigeorgica GUH-2 extract revealed an ion with *m/z* 868.3966 [M-2H+Ga]^+^ matching the predicted MS properties of brasilibactin A (see Fig. S21 at https://doi.org/10.5281/zenodo.3784407). Moreover, gene cluster and MS analysis of Nocardia gamkensis NBRC 108242 (subfamily VII) conform to the synthesis of nocobactin derivatives comprising a methyloxazole, an *N*-formyl group, and a central *gem*-dimethylated hydroxy fatty acid moiety. Such a core skeleton is known from formobactin and the amamistatins. Based on high-resolution MS (HRMS) and MS/MS spectra, the detected compound with *m/z* 810.3199 [M-2H+Ga]^+^ does indeed correlate with formobactin (see Fig. S12 at https://doi.org/10.5281/zenodo.3784407). Overall, we have identified candidate gene clusters for the biosynthesis of formobactin, the nocardimicins, and brasilibactin and provided the first evidence for their function. The pathways for these molecules have not been reported so far. Moreover, the production of these and the other known nocobactin-like siderophores, nocobactin NA and terpenibactin, corresponds to the assignment of their gene clusters to different subfamilies. Notably, the biosynthetic pathways clustered in subfamily VIII appear to produce variants of this structural class that have not been described before. The representative investigated, Nocardia rhamnosiphila NBRC 108938, accumulates a molecule likely containing a nonmethylated oxazoline, an *N*-formyl group, and a *gem*-dimethylated hydroxy fatty acid moiety. We are currently pursuing isolation and characterization of this new compound in detail. Another interesting finding are two metabolites from *N. gamkensis* NBRC 108242 which exhibit the characteristic MS fragmentation pattern of nocobactins but have a mass indicating the presence of a carboxyl group. The detection of MS fragments with *m/z* 458.0477, *m/z* 412.0432, *m/z* 171.0688, and *m/z* 145.0981 for parent ion *m/z* 840.2982 [M-2H+Ga]^+^ and fragments *m/z* 458.0450, *m/z* 412.0419, *m/z* 171.0480, and *m/z* 145.0894 for parent ion *m/z* 812.2634 [M-2H+Ga]^+^ implies intact phenyl methyloxazole, *N*-formylated *N*-hydroxylysine, and ε-caprolactam moieties. This suggests that it is the central hydroxy fatty acyl unit, which is modified by an additional carboxylic acid group. Such structural variations are known from carboxymycobactins, the extracellular version of mycobactins, which contain α,ω-dicarboxylic acids (53). Analogously, the two molecules detected may represent “carboxynocobactins,” the first examples of similarly oxidized nocobactin-like siderophores. We are currently further exploring this hypothesis.

Next, we wanted to validate our MS- and genomic data-based structure prediction strategy. We were particularly interested to examine molecular variations that are invisible to our MS/MS analysis design and are thus mainly deduced from gene cluster content. One such example is the location of an additional methylene unit (+14 Da) in fragment ions representing the western part of the molecule, e.g., in *m/z* 838.3512 [M-2H+Ga]^+^ from *N. alba* NBRC 108234 (see Fig. S23 at https://doi.org/10.5281/zenodo.3784407). In theory, this could derive either from the presence of a methyloxazoline or an *N*-acetyl group in the respective compounds. Therefore, we fed l-[^13^C_1_]serine to cultures of *N. alba* NBRC 108234 and analyzed the culture extracts by LC-MS. Detection of parent ion *m/z* 839.5489 [M-2H+Ga+1]^+^ and fragments *m/z* 413.2523 and *m/z* 441.2829 indicate the incorporation of l-serine into the oxazoline ring and, hence, the presence of an *N*-acetyl group (see Fig. S23 and S24 at https://doi.org/10.5281/zenodo.3784407). For mycobactin biosynthesis, it has been proposed that the oxazoline ring derives from demethylation of a methyloxazoline moiety (60). However, our data suggest that for nocobactin-type siderophores in *Nocardia* spp., the selected methylation of the oxazoline ring instead depends on the specificity of the respective A domain in the NbtF homolog toward threonine or serine.

### Identification of a mycobactin-type molecule produced by *N. araoensis* NBRC 100135.

We were not able to identify nocobactin-type molecules from Nocardia araoensis NBRC 100135 and N. takedensis NBRC 100417, although both have the biosynthetic capacity in the form of pathways belonging to subfamilies III and VII, respectively (see Table S18 at https://doi.org/10.5281/zenodo.3784407). This finding made us curious, and we reexamined the culture extracts of these strains to identify putative alternative siderophores. More precisely, we searched the respective MS spectra for molecular ions with a mass shift and isotope pattern that indicated the scavenging of Ga^3+^. Indeed, we found the ions of putative siderophores in the same mass range as that for the nocobactins, e.g., *m/z* 824.3353 [M-2H+Ga]^+^, *m/z* 840.3669 [M-2H+Ga]^+^, and *m/z* 854.3817 [M-2H+Ga]^+^. Intriguingly, MS/MS experiments produced signal spectra that would instead correlate with mycobactin-type molecules, wherein the long fatty acid moiety is *N-*acylated on the *N-*hydroxylysine instead of being incorporated by NbtB and NbtC in the polyketide extension comprising the ester linkage. To explore this possibility, we isolated a compound with the mass of 773.4569 Da from culture extracts of N. araoensis NBRC 100135. Following overnight incubation of this unknown putative mycobactin-like siderophore with Ga_2_(SO_4_)_3_ dissolved in methanol, we removed the residual solvent and resuspended it in CDCl_3_ for a suite of one-dimensional (1D) and 2D nuclear magnetic resonance (NMR) spectroscopic analyses. Structural assignment of the gallium-bound siderophore (Fig. 6; see Table S17 and Fig. S25 to S29 at https://doi.org/10.5281/zenodo.3784407) allowed us to identify key spin systems by total correlation spectroscopy (TOCSY): an *ortho*-disubstituted phenol, a serine-derived oxazoline, a linear saturated fatty acid, two *N-*hydroxylysines, and most notably, a β-hydroxybutyrate moiety. The presence of the relatively simple β-hydroxybutyrate spin system instead of the much more complex β-hydroxy fatty acid observed in the nocobactins further validated the prediction that this molecule is indeed a mycobactin-like siderophore. It was determined that the fatty acid was attached to the linear *N*-hydroxylysine side chain via key ^1^H-^13^C heteronuclear multiple bond correlation (HMBC) cross peaks from the same amide carbonyl carbon to both the amino acid ε-CH_2_ and the fatty acid α-CH_2_, further supporting the MS/MS fragmentation data regarding the location of this moiety. A direct NMR comparison of this novel metabolite to the most structurally similar molecules (mycobactin S or BE-32030 B from another *Nocardia* species [61]) was not performed due to the presence of a chelating Ga^3+^ ion within our analyses, while previous isolates contained the metal-free forms. One beneficial outcome of the addition of gallium is that it minimized rotomeric forms of this peptide and easily identified groups adjacent to metal-chelating moieties (the ε-CH_2_ groups of both *N*-hydroxylysines and the α-CH_2_ of the fatty acid) due to the strong diastereotopic shifts from chiral coordination to the metal ion. The nocobactin-type BGCs from *N. araoensis* NBRC 100135 and *N. takedensis* NBRC 100417 are genuine representatives of subfamilies III and VII, respectively. They are almost identical to the pathways of *N. farcinica* IFM 10152 and *N. gamkensis* NBRC 108242, respectively, the other investigated members of those clades. On the basis of these gene clusters, it is difficult to explain why they would support the production of mycobactins instead of nocobactins. We would speculate that for the biosynthesis of mycobactins in *Nocardia* spp., *trans*-encoded enzymes are recruited from other metabolic pathways, e.g., a long-chain fatty acid *N*-acyltransferase.

### Conclusion.

Our comparative genomic survey of the actinobacterial genus *Nocardia* revealed a highly diverse biosynthetic capacity for natural products which rivals better investigated genera such as *Streptomyces* and *Amycolatopsis*. Most of the GCFs identified by BGC similarity networks could not be correlated with known compounds, making *Nocardia* a promising source for the discovery of new drug leads. Other GCFs are widespread in or even constitutive for this genus, raising interesting questions about their role in bacterial physiology, ecology, and pathogenesis. By using the highly conserved biosynthetic pathway for nocobactin-type siderophores as a proxy, we sought to address the longstanding question of the relationship of BGC homology and chemical diversity. We could show that at least for this case, the BiG-SCAPE gene cluster subfamilies generated by a similarity cutoff of 0.7 encoded structurally distinct nocobactin derivatives. These congeners differed in oxidation state and the selected presence of various methyl groups. By using this approach, we were able to assign for the first time BGC candidates to the nocardimicins, formobactin, and brasilibactin. Moreover, we identified the *Nocardia* strains of subfamily VIII that likely produce so far undescribed nocobactin variants. Our study shows the successful linkage of comparative genomics data to chemical analytics data for refined chemical structure prediction. It exemplifies how multi-omics data can be implemented into drug discovery and aid dereplication efforts. With public repositories such as MassIVE and MetaboLights for MS/MS data and additional community tools, which provide links to genomic data (e.g., http://pairedomicsdata.bioinformatics.nl), this approach will be even more powerful in the future. Our results will thus facilitate future genome mining approaches and open the door for the targeted exploitation of the genus *Nocardia* for natural product discovery.

## MATERIALS AND METHODS

### Strains and growth conditions.

Chemicals, microorganisms, and molecular biological agents were purchased from standard commercial sources. Nocardia araoensis NBRC 100135, Nocardia cyriacigeorgica GUH-2, Nocardia takedensis NBRC 100417, *Nocardia* sp. strain Root136, Nocardia terpenica IFM 0406, Nocardia jejuensis NBRC 103114, Nocardia fusca NBRC 14340, Nocardia rhamnosiphila NBRC 108938, Nocardia vermiculata NBRC 100427, Nocardia gamkensis NBRC 108242, Nocardia elegans NBRC 108235, Nocardia seriolae DSM 44129, and Nocardia alba NBRC 108234 were obtained from the German Collection of Microorganisms and Cell Cultures (DSMZ). Strains were grown and maintained either on tryptic soy broth (TSB) (pancreatic digest of casein [17 g/liter], papaic digest of soybean [3 g/liter], sodium chloride [5 g/liter], dipotassium phosphate [2.5 g/liter], dextrose [2.5 g/liter]) purchased from Becton Dickinson or brain heart infusion (BHI), purchased from Sigma-Aldrich.

### *Nocardia* genomic data.

All *Nocardia* genome sequences used in this study were downloaded from the National Center for Biotechnology Information (NCBI) database or the Department of Energy (DOE) Joint Genome Institute – Integrated Microbial Genomes & Microbiomes (JGI IMG) database (62, 63). Genomes that consisted of more than 615 contigs were discarded from analysis. The completeness of the genomes were assessed using checkM (see Table S20 at https://doi.org/10.5281/zenodo.3784407) (64).

### Phylogenetic analysis (autoMLST).

A multilocus sequence analysis (MLSA) (65) was performed with autoMLST using the web-based application at http://automlst.ziemertlab.com/analyze# with the “denovo mode” and default settings (15). The resulting phylogenetic tree was visualized and modified in Geneious.

### *In silico* assessment of biosynthetic potential of *Nocardia* strains.

*In silico* analysis was performed with BLAST, antiSMASH 3.0 and 4.0 versions, and Geneious 9.1.8. BLASTP searches were performed using the NCBI Protein BLAST program against the nonredundant protein sequence database (66).

### BiG-SCAPE sequence similarity network.

All files were processed using antiSMASH 4.0 including the ClusterFinder border prediction algorithm (13, 25). The ClusterFinder border prediction algorithm was used to automatically trim BGCs where gene cluster borders were possible to predict. To the resulting 3,614 putative BGCs, 1,817 more were added from the MIBiG database as reference data (release 1.4, August 2018; see Fig. S31 at https://doi.org/10.5281/zenodo.3784407) (67). The final BGC set was analyzed using BiG-SCAPE version 201804 implemented in Python 2.7.13 (14). The “hybrids” mode, which allows BGCs with mixed annotations to be analyzed together, was enabled, and several cutoffs from 0.05 to 0.95 were tested. The program was run in both global and glocal alignment modes (see supplemental material at https://doi.org/10.5281/zenodo.3784407). Obtained sequence similarity matrices were then visualized using Cytoscape 3.6.1 (68).

### Production conditions.

Production of nocobactin- and mycobactin-like siderophores was achieved by using *Nocardia* mycelium from petri dishes (80 mm by 2 mm) containing BHI agar for preculture inoculation. *Nocardia* strains were cultured at 30°C and 200 rpm for at least 1 week. Five milliliters of preculture was then used to inoculate a main culture and shaken at 30°C at 200 rpm for at least 20 days. As production medium, a minimal medium (glycerol [20 ml/liter], l-asparagine [5 g/liter], KH_2_PO_4_ [5 g/liter or 50 g/liter] [pH 7.5]) was used as described previously (69). Chelex 100 sodium form, purchased from Sigma-Aldrich, was added, and the medium was filtered through Whatman no. 541 filter papers to remove Chelex. After autoclaving, media were supplemented with Zn^2+^ (0.5 μg/ml), Mn^2+^ (0.1 μg/ml), Mg^2+^ (0.04 mg/ml), and Fe^2+^ (0.05 μg/ml) or Fe^2+^ (2 μg/ml) for iron-deficient (main culture) or iron-sufficient (preculture) growth, respectively. All *Nocardia* strains were cultured in 300-ml precleaned Erlenmeyer flasks with 50 ml medium each at 30°C at 200 rpm. All glassware was precleaned by 2% (wt/vol) alcoholic KOH/8 N HNO_3_ treatments according to Ratledge and Winder (70),

### Extraction of mycobactin and nocobactin-like siderophores.

After at least 2 weeks of inoculation, mycelia were separated from the culture supernatant by centrifugation. Twenty-five milliliters of LC-MS grade methanol was added to the cell pellet. After sonication and centrifugation, the organic phase was isolated and evaporated under reduced pressure. The resulting crude extracts were dissolved in methanol (MeOH). To obtain siderophores bound to gallium, a solution of gallium sulfate (20 mg in 30 ml MeOH) was prepared. Ten milliliters was added to crude extracts and evaporated under reduced pressure.

### Isolation and purification of a mycobactin-like siderophore by HPLC.

For the isolation of a mycobactin-like siderophore from Nocardia araoensis NBRC 100135, an Agilent Technologies ProStar 410 Auto Sampler equipped with a Phenomenex Luna 5-μm C18(2) 100-Å high-performance liquid chromatography (HPLC) column (100 by 21.2 mm) was used with an Agilent Technologies 440-LC fraction collector and a flow rate of 10 ml/min with water plus 0.02% formic acid as solution A and methanol with 0.02% formic acid as solution B (see Table S1 at https://doi.org/10.5281/zenodo.3784407). Fractions generated were subjected to an Agilent Technologies 1100 HPLC system equipped with a Phenomenex Luna 5-μm C8(2) column (250 by 10 mm; 100 Å) at a flow rate of 3.5 ml/min (see Table S2 at https://doi.org/10.5281/zenodo.3784407). The mycobactin-like siderophore with the mass of 840.367 [M-2H+Ga]^+^ from N. araoensis NBRC 100135 eluted after 15.6 min. The peak was collected and dried to yield an orange-to-red oil.

### Isolation of gDNA.

To isolate genomic DNA (gDNA), *Nocardia* precultures from either BHI or TSB agar plates were used to inoculate 50 ml of liquid TSB or BHI medium and cultivated for at least 2 weeks at 30°C and 200 rpm. Cells were harvested, and genomic DNA was isolated from *Nocardia* with standard protocols for *Streptomyces* by phenol-chloroform extraction. Protocols were followed as described in reference 71 with additional treatment of 40 mg proteinase K and subsequent incubation at 60°C for at least 30 min, but no longer than 1 h, after the addition of sodium dodecyl sulfate (SDS).

### 16S rRNA gene analysis.

To rule out contamination of Nocardia araoensis NBRC 100135 production cultures, gDNA was isolated from respective precultures. The 16S rRNA gene was amplified by PCR with the 27F and 1492R universal eubacterial 16S rRNA gene primer set and high-fidelity Q5 polymerase (see Table S16 at https://doi.org/10.5281/zenodo.3784407). PCR products were analyzed by gel electrophoresis. The Zero Blunt PCR cloning kit was used to clone PCR fragments, and 20 positive clones were sequenced. A BLASTn search against the NCBI nucleotide database showed “Nocardia araoensis NBRC 100135” as the first hit for all 20 clones. In addition, production cultures were examined visually by microscopy and inoculation of selective media. No contamination could be observed.

### Mycobactin-like siderophore structure elucidation.

NMR solvents were obtained from Sigma-Aldrich and used without further purification.

For NMR analysis, 1.5 mg gallium sulfate was dissolved in 3 ml deuterated methanol and then incubated with the mycobactin-like siderophore overnight. The solvent was removed *in vacuo*, and the Ga-bound species was resuspended in CDCl_3_ for NMR analyses. Chemical shift signals (δ) are reported in parts per million and are referenced to the internal chloroform signal (δ = 7.26 ppm for ^1^H; δ = 77.2 ppm for ^13^C).

### LC-HRMS and MS/MS analysis.

High-resolution mass spectra (HRMS) were collected on an Agilent 6530 Accurate-Mass Quadrupole Time-of-flight (QTOF) LC-MS instrument (Agilent Technologies) with an electrospray ionization (ESI) interface. Ten to 15 μl of each crude extract was injected onto a reversed-phase C_18_ column [Phenomenex Luna 5-μm C18(2) column [4.6 × 150 mm; 100 Å]) on a 1260 Infinity LC-System (Agilent Technologies) using a flow rate of 0.75 ml/min to separate the analytes. MS analysis was performed by ESI in positive ionization mode (50 eV). A gradient from 80% solvent A (water with 0.1% formic acid) to 97% solvent B (acetonitrile [ACN] with 0.06% formic acid) was applied.

### Global Natural Products Social Molecular Networking (GNPS) molecular MS/MS network.

A molecular network was created to simplify the discovery of new nocobactin and mycobactin derivatives using the online workflow at Global Natural Products Social Molecular Networking (GNPS) (59). High-resolution LC-MS data were converted to .mzxml format using MSConvert version 3.0.18353-2de7e414e. The data were clustered with MS-Cluster with a parent ion mass tolerance of 0.04 Da and a fragment ion mass tolerance of 0.04 Da to create consensus spectra. Networks were created where edges were filtered to have a cosine score above 0.6 and more than three matched peaks. A peak intensity of 50 was set as minimum peak intensity. The resulting molecular network was visualized in Cytoscape 3.6.1 (68). The MS/MS data were deposited in MassIVE (MSV000084771) and linked with the genomic data at the iOMEGA Pairing Omics Data Platform (http://pairedomicsdata.bioinformatics.nl/projects).

### Stable isotope labeling.

For feeding experiments with l-[1-^13^C]serine, labeled substrates were added after 2 days of cultivation in a 5 mM final concentration, respectively. High-resolution mass spectra for feeding experiments as well as nocobactin-like siderophore metabolomics data from Nocardia cyriacigeorgica GUH-2 were recorded on an HR-ESI-TOF-MS maXis 4G mass spectrometer (Bruker Daltonik GmbH, Bremen, Germany).

### Statistical analysis.

BiG-SCAPE annotations were used to compute the BGC class frequency for all the *Nocardia* genomes. The statistical significance for differences in the BGC class frequency between group 1 pathogens and other *Nocardia* genomes was calculated using *t* test in Rstudio version 1.1.383. Boxplots were generated in Rstudio using the package ggpubr version 0.2.5 and ggplot2. BiG-SCAPE presence/absence matrix containing information on gene cluster families (GCFs) across all the *Nocardia* genomes was used to perform principal-component analysis (PCA) using ClustVis web tool.
